# Perceived parental competence and mental health in school-aged adolescents in Colombia: evidence from a well-being and risk approach

**DOI:** 10.3389/frcha.2026.1890077

**Published:** 2026-07-13

**Authors:** Felipe Agudelo-Hernández, Marcela Guapacha-Montoya, Andrés Camilo Delgado-Reyes, Lorena Aguirre-Aldana

**Affiliations:** 1 Medicine Program, Faculty of Health Sciences, University of Manizales, Manizales, Colombia; 2 Medicine Program, Faculty of Health Sciences, University of Caldas, Manizales, Colombia; 3 Psychology Program, Faculty of Social Sciences and Humanities, University of Manizales, Manizales, Colombia

**Keywords:** adolescent, depression, mental health, parental competence, parenting, perceived parental competence, resilience, psychological

## Abstract

**Background:**

This study examined adolescent-reported perceived parental competence as a relational indicator associated with mental health and well-being in Colombian adolescents. Perceived parental competence was defined as youths' appraisal of caregivers' support, responsiveness, monitoring, communication, and boundary-setting, and was modeled as the dependent variable rather than as a causal determinant of adolescent outcomes.

**Methods:**

A quantitative, cross-sectional, analytical study was conducted with 1,005 adolescents from three convenience-selected school settings representing urban Manizales, semi-urban Anserma, and rural Bahía Solano, Colombia. Participation was voluntary and exceeded 95% of eligible students in the selected schools. All measures were administered in Spanish and completed by adolescents as self-report questionnaires. The PANSI was operationalized as a risk-oriented suicidal ideation score by combining negative ideation items with reverse-coded protective ideation items. The CRAFFT-related variable was analyzed as a continuous substance-use screening score, not as a categorical risk classification.

**Results:**

Perceived parental competence was positively correlated with life satisfaction (r = 0.362, *p* < 0.001) and resilience (r = 0.283, *p* < 0.001) and negatively correlated with depressive symptoms (r = -0.278, *p* < 0.001) and risk-oriented suicidal ideation (r = −0.354, *p* < 0.001). The continuous substance-use screening score showed a small positive bivariate association with perceived parental competence (r = 0.114, *p* < 0.001), but it was not retained in the final regression model. The final model retained life satisfaction, origin, resilience, risk-oriented suicidal ideation, depressive symptoms, and frustration tolerance, explaining 26.4% of the variance in perceived parental competence.

**Conclusions:**

Youth-perceived parental competence was mainly associated with higher life satisfaction, greater resilience, fewer depressive symptoms, and lower risk-oriented suicidal ideation. The CRAFFT-related score should be interpreted only as a continuous screening indicator derived from the available questionnaire coding, not as a validated categorical classification of substance-use risk. The cross-sectional design precludes causal interpretation, but the findings support assessing adolescent perceptions of parental responsiveness and monitoring as relational indicators in school and community mental health strategies.

## Introduction

Mental health problems in children and adolescents are a growing public health priority because of their frequency, early onset, and cumulative effects on development, educational attainment, social relationships, and later functioning. Globally, one in seven adolescents aged 10–19 years is estimated to live with a mental disorder, and anxiety, depression, and behavioral disorders are among the leading causes of illness and disability during this stage of life (1). Consistently, the Global Burden of Disease Study estimated that, in 2019, approximately 293 million people aged 5–24 years were living with at least one diagnosable mental disorder, with a substantial burden in years lived with disability (2).

In Latin America and Colombia, adolescent mental health must be understood in relation to social inequalities, exposure to violence, barriers to specialized care, academic pressure, post-pandemic family strain, and territorial vulnerability (3–6). These conditions are relevant because they operate through family, school, and community environments and may influence how adolescents interpret protection, monitoring, communication, and emotional availability at home (3, 4, 7–10). For this reason, the present study treats perceived parental competence as a contextualized relational appraisal rather than as an isolated parental attribute or a direct causal determinant of adolescent mental health.

Perceived parental competence refers to adolescents' appraisal of their parental figures' ability to support, protect, guide, communicate, set boundaries, respond to developmental needs, and foster autonomy in an age-appropriate manner. This construct differs from externally observed parenting practices and from parental self-efficacy reported by mothers, fathers, or caregivers. Throughout this manuscript, perceived parental competence refers specifically to adolescents' reports of parental competence and should not be interpreted as parents' self-assessment or as an observational measure of parenting quality.

Recent evidence indicates that adolescents' perceptions of parental support, supervision, autonomy support, and emotional availability are associated with psychological adjustment, life satisfaction, self-regulation, school adjustment, lower loneliness, and fewer emotional symptoms (2, 7–9). Psychometric work on perceived parental competencies in Latin American adolescents also supports the relevance of assessing this construct from adolescents' own perspective (11). In the region, evidence on attachment, parental sensitivity, and family relationships supports the relevance of relational approaches to development, although available studies remain concentrated in urban and middle-class samples, limiting their generalizability to more socially and territorially diverse contexts (12).

During adolescence, parental influence depends not only on adult behavior but also on how adolescents interpret that behavior within the emotional climate and relational history of the family. Support, autonomy, recognition, non-intrusive guidance, and emotional regulation become increasingly salient because adolescents seek both protection and independence during this developmental period (13, 14). Accordingly, perceived parental competence can be located within adolescents' proximal relational environments and examined in relation to well-being, symptom, school, and contextual indicators.

Adolescent mental health should not be defined solely by the absence of symptoms. Contemporary two-factor models propose that mental health includes two related but distinct dimensions: a negative dimension, associated with psychological distress, symptoms, risk, and dysfunction, and a positive dimension, linked to well-being, personal resources, adaptive functioning, and life satisfaction (15, 16). This perspective avoids reducing adolescent mental health to the presence or absence of psychopathology and allows the identification of more complex psychosocial profiles, including adolescents with low symptoms but limited well-being, moderate symptoms but relevant protective resources, or high symptoms and low well-being.

Applied to perceived parental competence, the dual-factor model provides a framework for examining associations with both symptomatic indicators and positive dimensions of mental health. Greater perceived parental competence may be associated with stronger personal resources, including resilience, problem-solving skills, frustration tolerance, and subjective well-being, as well as with lower emotional distress and lower exposure to risk behaviors (14–17). This framework is particularly relevant for preventive and mental health promotion strategies in settings where specialized care is limited or delayed (5).

Resilience, frustration tolerance, problem-solving, and subjective well-being represent psychological and relational resources that may support adaptation to everyday demands and adverse events. By contrast, depressive symptoms, substance-use screening scores, suicide-related risk, academic difficulties, and environmental distress represent relevant indicators of symptoms, risk, or functional vulnerability during adolescence. Eco-distress and solastalgia have also gained relevance in adolescent mental health research because they describe forms of distress related to the loss, threat, or transformation of the inhabited environment, particularly in populations exposed to environmental degradation, territorial insecurity, or climate anxiety (18–20).

Several explanatory models help locate perceived parental competence within adolescent mental health. From self-determination theory, parental figures may support psychological adjustment when they promote autonomy, competence, and relatedness, whereas practices perceived as psychological control, emotional invalidation, inconsistency, or rejection may frustrate these needs (21). From ecological systems theory, the family, school, and peer group are proximal microsystems embedded in broader social and cultural contexts (22). Integrative and dimensional models of parenting further distinguish warmth, responsiveness, demandingness, structure, supervision, and control, whose meaning and effects may depend on how adolescents perceive them and on the cultural context in which they occur (13, 23–26).

Culturally sensitive parenting and family-based approaches are relevant to this framework because local caregiving norms, household constraints, community resources, and expectations about autonomy and authority shape how parental competence is interpreted (5, 6). In low-resource and culturally diverse settings, parental monitoring may be experienced as care, control, protection, mistrust, or necessary supervision, depending on the surrounding social and territorial conditions. This literature therefore supports examining adolescent-reported perceived parental competence as a relational construct associated with both well-being resources and psychosocial risk indicators.

**Figure 1:**
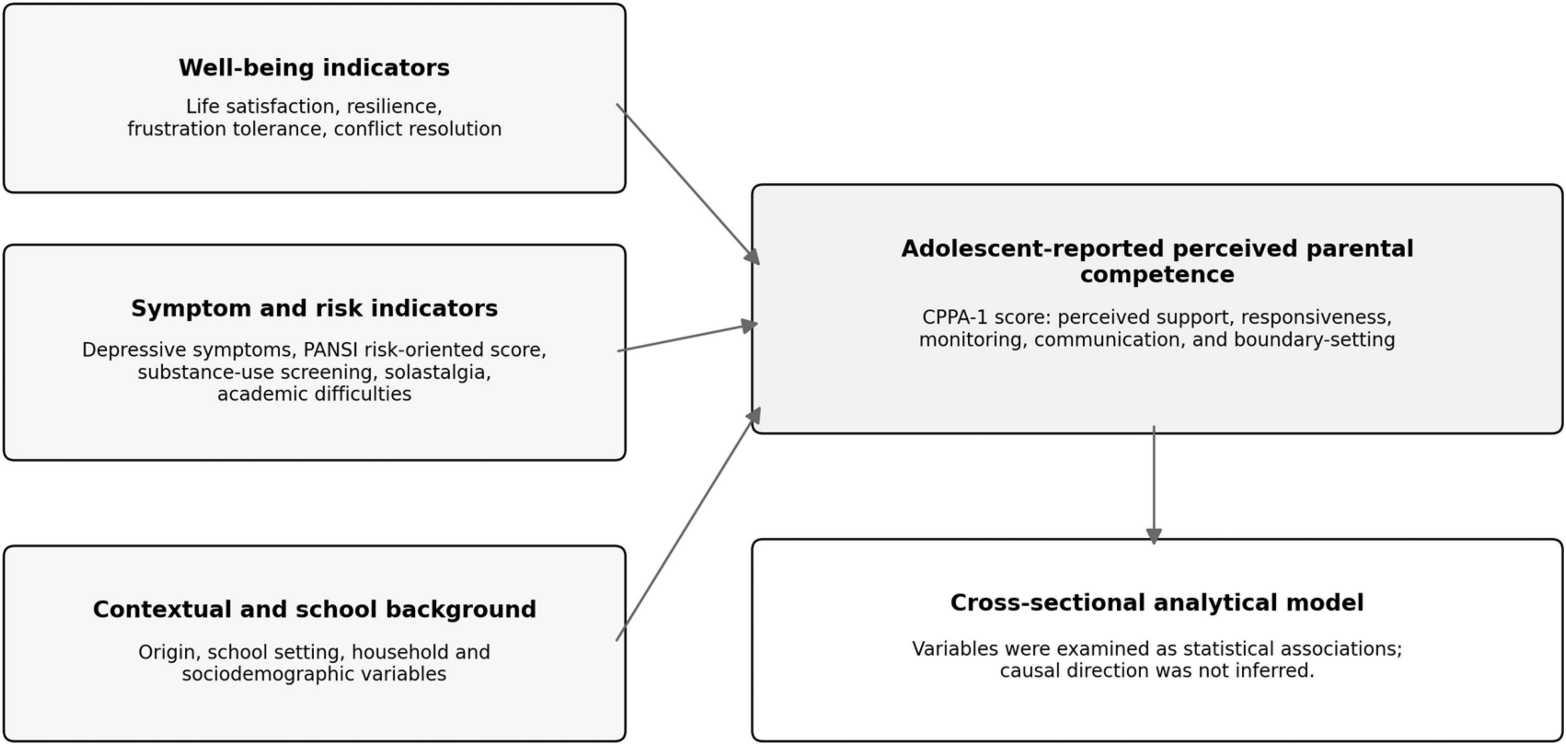
Conceptual framework.

Within this framework, perceived parental support, availability, sensitivity, open communication, and protective supervision may strengthen emotional security, help-seeking confidence, emotion regulation, conflict resolution, self-efficacy, connectedness, and subjective well-being (8, 10). Conversely, lower perceived parental competence may coexist with helplessness, isolation, family conflict, emotional avoidance, reduced help-seeking, and greater reliance on maladaptive coping strategies. These mechanisms informed the study hypotheses but were not treated as causal pathways, given the cross-sectional design (Figure 1).

Examining these associations in Colombia has theoretical, clinical, and public health relevance. Much of the evidence on parenting, well-being, and adolescent mental health comes from high-income countries and may not fully reflect the social, family, educational, and territorial conditions of Latin America and the Global South (5, 6, 12). In Colombia, inequality, barriers to psychological care, community violence, academic pressure, changing family structures, economic uncertainty, and environmental stressors may contribute to cumulative psychosocial exposure, while families may function either as sources of protection and belonging or as contexts of conflict, neglect, control, or invalidation.

The gap addressed by this study is not simply the limited availability of Colombian data. More specifically, there is limited evidence linking adolescent-reported family appraisal with positive and symptomatic mental health indicators across heterogeneous school contexts. By centering adolescents' perspectives, this study examines how perceived support, responsiveness, monitoring, and relational availability covary with resilience, life satisfaction, depressive symptoms, risk-oriented suicidal ideation, substance-use screening, environmental distress, and school-related functioning.

This study aimed to examine the association between perceived parental competence and positive, symptomatic, school-related, and contextual indicators in school-attending adolescents in Colombia. Resilience, frustration tolerance, problem-solving, and subjective well-being were included as well-being indicators, whereas depressive symptoms, learning-related difficulties, a continuous substance-use screening score, environmental distress, and risk-oriented suicidal ideation were included as indicators of distress or psychosocial risk. We hypothesized that higher perceived parental competence would be positively associated with well-being indicators and negatively associated with symptomatic or risk-oriented indicators of mental health, with all associations interpreted in non-causal terms because of the cross-sectional design.

## Methods

### Study design

A quantitative, cross-sectional, and analytical study was conducted to examine the psychosocial, school, family, and health factors associated with perceived parental competence in school-aged adolescents in Colombia. The cross-sectional design allowed for the single-point measurement of perceived parental competence, well-being indicators, mental health symptoms, school variables, and sociodemographic characteristics of the participants. Due to the nature of the design, the results were interpreted as statistical associations and not as causal relationships. To align the stated aims with the statistical model, perceived parental competence was specified as the outcome of interest. The study therefore examined which well-being, symptom, school, and contextual variables were associated with adolescents' perception of parental competence at one point in time.

The sample included adolescents from urban, semi-urban, and rural contexts; however, the study did not aim to compare perceived parental competence across these three contexts. The inclusion of participants from different regions was intended to increase sample heterogeneity and capture greater social, family, school, and contextual variability. For this reason, geographic origin was considered a contextual variable within the analysis and not the primary comparative axis of the study.

### Participants

The study population consisted of school-aged adolescents from Colombian educational institutions located in urban, semi-urban, and rural areas. A census sampling strategy was used, inviting all eligible students to participate. Adolescents were included only if they had obtained informed consent from their caregivers and provided informed assent. Participation was voluntary, confidential, and had no academic, disciplinary, or institutional consequences. Records lacking consent or assent, questionnaires with insufficient information to calculate the main variables, and cases with critical inconsistencies in identification, institution, or contextual classification variables were excluded. The original protocol reported a final sample of 1,005 school-aged adolescents, with data collection coordinated with educational institutions and local health departments.

The schools and municipalities were selected by convenience to increase contextual heterogeneity rather than to obtain a nationally representative sample. All eligible students in the selected schools were invited to participate, and the final sample consisted of adolescents who voluntarily decided to participate after caregiver consent and adolescent assent. Participation exceeded 95% of the potentially eligible students in the selected institutions. The participating schools were the main secondary schools in their respective study sites: Bahía Solano, Chocó, was treated as the rural site and has an estimated 2026 population of approximately 10,943 inhabitants; Anserma, Caldas, was treated as the semi-urban site and has an estimated 2026 population of approximately 37,352 inhabitants; and Manizales, Caldas, was treated as the urban site and has an estimated 2026 population of approximately 475,690 inhabitants, according to National Administrative Department of Statistics municipal population projections (27). Indigenous self-identification in the sample varied by site: approximately 10.0% in Bahía Solano, 7.0% in Anserma, and 0.3% in Manizales. These contextual data are provided to clarify sample selection and generalizability; they should not be interpreted as population-representative estimates.

### Variables and instruments

The dependent variable was perceived parental competence, assessed using the Parental Competence Perception Questionnaire for Adolescents (CPPA-1) (28). This instrument is designed for adolescents between 12 and 17 years old and assesses their perception of their primary caregivers' parental competence. It includes dimensions related to attachment, involvement, parental monitoring, and adolescent responses to parenting practices. It consists of 48 Likert-type items and, in its design and validation process with the Colombian population, demonstrated high internal consistency, with a Cronbach's alpha of.961. The scale is interpreted directly: higher scores indicate greater perceived parental competence.

The broader CPPA-1 includes multiple domains of adolescent-perceived parental competence. In the present analysis, the dependent variable was the five-item perceived parental competence score available in the study questionnaire and included in the final model. These items assessed adolescents' appraisal of caregiver responsiveness, support, monitoring, and relational availability; scores ranged from 0 to 25, higher scores indicated higher perceived parental competence, internal consistency was good in the present sample (Cronbach's alpha = 0.861), and no items were reverse-coded.

Depressive symptoms were assessed using the Patient Health Questionnaire-9 (PHQ-9), a nine-item scale that explores depressive symptoms during the previous two weeks, including anhedonia, depressed mood, sleep and appetite disturbances, fatigue, feelings of worthlessness, difficulty concentrating, psychomotor changes, and suicidal ideation or self-harm. In Colombia, it has been reported evidence of validity for depression screening in primary care, with a Cronbach's alpha of.80, McDonald's omega of.81, and an area under the curve of.92 (29).

Suicide-related ideation was assessed using the Positive and Negative Suicide Ideation Inventory (PANSI), a 14-item self-report instrument that includes two conceptually distinct components: negative suicidal ideation and positive ideation or reasons for living (30). Because these components have opposite valence, the present analyses did not use the untransformed raw total score. A risk-oriented PANSI score was computed by summing the eight negative ideation items (items 1, 3, 4, 5, 7, 9, 10, and 11) and the six positive ideation items after reverse coding (items 2, 6, 8, 12, 13, and 14), so that higher values consistently indicated higher suicide-related risk and lower protective ideation. Scores were computed when at least 12 of 14 items were valid and prorated to the 14-item metric. The resulting score had a possible range of 14–70, and internal consistency for the transformed risk-oriented score was good in the present sample (Cronbach's alpha = 0.870).

Resilience was measured using the 10-item Connor-Davidson Resilience Scale, CD-RISC-10, which assesses perceived coping, adaptation, and recovery capacity in the face of adversity (12). Psychometric evidence exists for this scale in the adult population in Colombia (31). Since this validation was not specifically performed in adolescents, the original study estimated internal consistency in the adolescent sample, with a Cronbach's alpha of.92. This result supports its analytical use in the sample, although this psychometric limitation should be acknowledged.

Psychoactive substance use was assessed using CRAFFT-related screening items for adolescents. The present analyses used a continuous derived screening score rather than the standard binary CRAFFT risk classification. This score was calculated as the sum of the seven substance-use screening items available in the dataset. Higher values indicate greater endorsement of substance-related experiences within the questionnaire coding used in this survey. The validated CRAFFT cutoff of 2 or higher was not applied because the available database contained an extended ordered-response coding rather than the conventional 0/1 CRAFFT item scoring required to form the standard 0–6 total score (32). Therefore, this variable is reported as a continuous substance-use screening score and should not be interpreted as a diagnosis, a substance-related disorder, or a standard CRAFFT risk category.

Life satisfaction was assessed using the Satisfaction With Life Scale (SWLS), a five-item scale that measures individuals' global cognitive appraisal of their lives (33). In Colombia, the scale has shown good psychometric properties, a unidimensional structure, factorial equivalence with Spanish samples, and adequate internal consistency, with an overall Cronbach's alpha of.89 (34). A children's version has also shown a unidimensional structure and acceptable internal consistency in Colombian participants aged 10–14 years (35).

School-based socioemotional competencies were assessed using brief versions of the Frustration Tolerance and Conflict Resolution questionnaires from the Social Competence and School Health program. Frustration tolerance assesses recognition of frustration, emotional control, adherence to rules, and search for alternatives. Conflict resolution assesses conflict identification, negotiation, empathy, and coping. These scales were designed for school settings and have prior evidence of use and validation among students in Bogotá (28).

School-related and functional variables were also collected through self-reported questions, including enjoyment of school, presence of friends at school, bullying, difficulties understanding instructions, letter confusion when reading or writing, and difficulties with mathematical processes. These variables were included as indicators of school functioning and academic adjustment, rather than as clinical or neuropsychological diagnoses. Sociodemographic and contextual variables were also included, such as age, sex, grade level, origin, household composition, ethnicity, perceived physical health, and perceived mental health. All questionnaires were administered in Spanish. The research team provided standardized instructions and clarification of item wording when requested, without influencing participants' responses. All variables analyzed in this study were reported by adolescents themselves. No parent-reported or teacher-reported measures were used in the statistical analyses. This point is now stated explicitly to avoid confusion between perceived parental competence and parents' own perceptions of their competence.

### Procedure

Data collection was conducted between August 2025 and February 2026 in school settings, in coordination with the participating educational institutions and the corresponding health departments. Before the instruments were administered, the study objective was presented to school administrators, teachers, caregivers, and students. Participants were informed that participation was voluntary, that responses would remain confidential, and that students could withdraw at any time without academic or institutional consequences. Caregivers provided written informed consent, and adolescents provided informed assent. Questionnaires were administered in school spaces arranged to ensure privacy, facilitate understanding of the items, and provide support from the research team when needed. Teachers, administrators, and other school authority figures did not have access to individual responses. As an additional ethical safeguard, the study included school-based and intercultural mental health support pathways, coordinated with psychosocial teams and local health departments. This component was not evaluated as an intervention; rather, it served as an ethical and community-based mechanism to activate care pathways when emotional symptoms, suicidal ideation, substance use, or other relevant psychosocial needs were identified.

### Statistical analysis

The database underwent a cleaning process that included duplicate review, verification of valid ranges, identification of impossible values, assessment of inconsistencies between age and grade level, removal of records without consent or assent, calculation of missing data by participant and by scale, and evaluation of potentially invalid response patterns. Means and standard deviations were calculated for continuous variables, whereas absolute frequencies and percentages were calculated for categorical variables. Internal consistency of the scales was assessed using Cronbach's alpha when appropriate.

Missing data were handled using available-case analyses for descriptive statistics and listwise deletion for regression models. No statistical imputation was performed. Cases were excluded when consent or assent was absent, when a questionnaire lacked sufficient information to compute the main scale totals, or when critical identification/contextual inconsistencies were detected. Range checks were also conducted.

For the bivariate analysis, Pearson correlation coefficients were estimated between perceived parental competence and sociodemographic, school-related, well-being, and mental health symptom variables. Subsequently, a multiple linear regression model was estimated with perceived parental competence as the dependent variable. Model assumptions were assessed through residual analysis, collinearity statistics, tolerance and variance inflation factor, and the Durbin-Watson statistic. The final model included life satisfaction, origin, resilience, the risk-oriented PANSI score, depressive symptoms, and frustration tolerance as predictors.

### Ethical considerations

The study was conducted in accordance with ethical principles for research involving human participants of the Declaration of Helsinki, with particular attention to the protection of children and adolescents. The protocol received institutional ethics approval by the Bioethics Committee of the Caldas Territorial Directorate and the University of Manizales (act CB_04-2025). Participation was voluntary and was preceded by informed consent from caregivers and informed assent from adolescents. Participants were informed that they could withdraw from the study at any time, without academic, disciplinary, institutional, or service-access consequences. Information was handled confidentially, and identification codes were used to protect students' identities. The study incorporated criteria for cultural safety, recognizing that the relationship with territory, the experience of environmental transformation, and the ways distress is expressed may vary among adolescents from urban, semiurban, rural, and intercultural contexts. Therefore, the instruments were administered using understandable language, with support when needed, and with respect for community and territorial particularities.

## Results

### Sample characteristics

The final analytical sample consisted of 1,005 school-aged adolescents from Colombia. After range checks, valid ages ranged from 11 to 19 years (M = 14.34, SD = 1.78). The descriptive profile showed substantial variation in well-being, symptom, school, and contextual indicators. For PANSI, descriptive statistics correspond to the risk-oriented score computed from negative ideation items and reverse-coded protective ideation items. For CRAFFT, descriptive statistics correspond to the continuous substance-use screening score derived from the available questionnaire coding. Table 1 summarizes the main continuous measures with observed ranges, as well as sociodemographic, school, and household characteristics of the analytical sample.

**Table 1:** Descriptive and sociodemographic data.

Variable	Mean	Standard Deviation	Range
PHQ-9 - Depression	9.34	7.354	0–36
Frustration Tolerance (FT)	10.02	2.42	0–16
Conflict Resolution (CR)	9.97	2.425	0–16
Perceived Parental Competence (CPPA)	20.13	5.104	0–25
Resilience	27.43	11.342	0–50
Solastalgia (BSS)	12.82	7.912	0–45
Continuous substance-use screening score (CRAFFT-related)	16.40	6.594	7–28
Satisfaction With Life Scale (SWLS)	23.75	7.89	0–35
Risk-oriented suicidal ideation (PANSI)	25.65	9.486	14–70

	Frequency	%	
Origin			
Urban	617	61.4	
Semi-urban	166	16.5	
Rural	222	22.1	
Ethnicity			
Indigenous	168	17.71	
Afro	250	24.87	
None	587	57.41	
Gender			
Male (Cisgender/Transgender)	527	52.4	
Female (Cisgender/Transgender)	468	46.6	
Non-binary/Gender Diverse	10	1	
Education level			
Fifth	6	.6	
Sixth	176	17.5	
Seventh	136	13.5	
Eighth	160	15.9	
Ninth	252	25.1	
Tenth	124	12.3	
Eleventh	134	13.3	
Twelfth	17	1.7	
Who do you live with?			
I live alone	334	33.2	
Mother	165	16.4	
Parents	255	25.4	
Grandparents	37	3.7	
Other relatives	90	9	
Father	5	0.5	
I prefer not to answer	106	10.5	
Other`	1	0.1	
Single father	12	1.2	
Religion			
None	29	7.8	
Catholic	337	90.6	
Christian	6	1.6	
Physical health			
Very poor	51	5.1	
Poor	103	10.2	
Fair	168	16.7	
Good	520	51.7	
Very good	163	16.2	
Mental health			
Very poor	49	4.9	
Poor	145	14.4	
Fair	192	19.1	
Good	481	47.9	
Very good	138	13.7	
Do you enjoy school?			
Yes	501	49.9	
No	398	39.6	
I prefer not to answer	106	10.5	
Bullying	Mean	SD	
Yes	280	27.9	
No	675	67.2	
I prefer not to answer	50	5	
Difficulties with math processes			
Yes	296	29.5	
No	680	67.7	
I prefer not to answer	29	2.9	
Difficulties understanding instructions			
Yes	291	29.0	
No	693	69.0	
I prefer not to answer	21	2.1	
Confusing letters when writing or reading			
Yes	230	22.9	
No	764	76.0	
I prefer not to answer	11	1.1	

Bivariate correlations showed that perceived parental competence was significantly associated with several dimensions of well-being, symptoms, risk screening, and school functioning. The strongest positive association was observed with life satisfaction (r = 0.362, *p* < 0.001), followed by resilience (r = 0.283, *p* < 0.001), conflict resolution (r = 0.143, *p* < 0.001), and frustration tolerance (r = 0.126, *p* < 0.001). These results indicate that adolescents with higher perceived parental competence tended to report more favorable subjective well-being and stronger coping or socioemotional resources.

Perceived parental competence was negatively associated with the risk-oriented PANSI score (r = −0.354, *p* < 0.001) and depressive symptoms (r = −0.278, *p* < 0.001). This pattern indicates that adolescents who perceived greater parental competence tended to report lower suicide-related risk and fewer depressive symptoms. A negative association was also observed with origin (r = −0.234, *p* < 0.001), which should be interpreted according to the coding of urban, semi-urban, and rural contexts rather than as a direct territorial comparison.

Smaller positive associations were identified between perceived parental competence and substance-use screening (r = 0.114, *p* < 0.001), bullying (r = 0.118, *p* < 0.001), solastalgia (r = 0.106, *p* = 0.001), difficulties in mathematics (r = 0.092, *p* = 0.004), difficulties understanding instructions (r = 0.085, *p* = 0.007), and perceived mental health (r = 0.082, *p* = 0.009). Perceived physical health (r = 0.059, *p* = 0.060) and letter confusion when reading or writing (r = 0.045, *p* = 0.152) were not statistically significant.

### Linear regression model for perceived parental competence

A multiple linear regression model was estimated with perceived parental competence as the dependent variable. The final model included life satisfaction, origin, resilience, the risk-oriented PANSI score, depressive symptoms, and frustration tolerance as predictors. The model yielded R = 0.514, R² = 0.264, and adjusted R² = 0.259, indicating that the set of predictors explained 26.4% of the variance in perceived parental competence. The standard error of the estimate was 4.079, and the Durbin-Watson statistic was 1.909, consistent with no relevant autocorrelation in the residuals. Collinearity diagnostics showed adequate values, with VIF values ranging from 1.057 to 1.418. Variables evaluated but not retained in the final model included sex, age, grade level, grade repetition, perceived physical health, perceived mental health, enjoyment of school, friends at school, bullying, academic support, difficulty understanding instructions, letter confusion, difficulties in mathematics, conflict resolution, solastalgia, and the continuous substance-use screening score (Table 2).

**Table 2:** Regression model for perceived parental competence.

Predictor retained in final model	B	SE	Standardized beta	t	p	VIF
Life satisfaction (SWLS)	0.125	0.020	0.203	6.247	< 0.001	1.341
Origin	−1.118	0.171	−0.193	−6.535	< 0.001	1.103
Resilience	0.081	0.013	0.188	6.289	< 0.001	1.133
Risk-oriented suicidal ideation (PANSI)	−0.075	0.017	−0.150	−4.498	< 0.001	1.418
Depressive symptoms (PHQ-9)	−0.057	0.021	−0.087	−2.684	0.007	1.333
Frustration tolerance	0.161	0.058	0.080	2.764	0.006	1.057

Dependent variable: perceived parental competence. R = 0.514, R² = 0.264, adjusted R² = 0.259, standard erro*r* = 4.079, Durbin-Watson = 1.909. Coefficients are displayed for predictors retained in the final model. The PANSI predictor was scored as a risk-oriented suicidal ideation indicator; the continuous CRAFFT-related score was evaluated but not retained in the final model.

**Figure 2:**
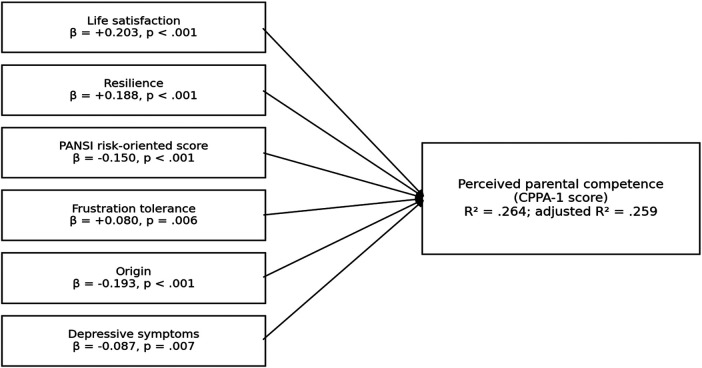
Final multiple linear regression model for perceived parental competence.

The final model figure presents the retained predictors, standardized coefficients, and explained variance. This visual summary complements Figure 2 by making the direction of each association explicit. It distinguishes positive coefficients for life satisfaction, resilience, and frustration tolerance from negative coefficients for origin, depressive symptoms, and risk-oriented suicidal ideation.

## Discussion

This study analyzed the association between perceived parental competence and a set of positive and symptomatic mental health indicators in Colombian adolescents. Resilience, frustration tolerance, conflict resolution, and life satisfaction were included as dimensions of well-being, whereas depressive symptoms, academic difficulties, environmental distress, a continuous substance-use screening score, and risk-oriented suicidal ideation were included as indicators of distress or psychosocial risk. The findings supported the main hypothesis: perceived parental competence was positively associated with well-being and socioemotional resources and negatively associated with depressive symptoms and risk-oriented suicidal ideation.

The pattern of associations was clearer after the PANSI was operationalized in a way that respected the opposite valence of its components. Frustration tolerance and conflict resolution showed small positive bivariate associations with perceived parental competence, and frustration tolerance remained in the adjusted model. Solastalgia also showed a small positive bivariate association, suggesting that environmental distress may coexist with relational resources and should not be interpreted as the absence of perceived family support. In contrast, risk-oriented suicidal ideation showed a negative association with perceived parental competence in both bivariate and adjusted analyses.

More specifically, the adjusted model identified a coherent pattern: perceived parental competence was higher among adolescents reporting greater life satisfaction, greater resilience, and higher frustration tolerance, and lower among those with more depressive symptoms and higher risk-oriented suicidal ideation. The association with origin indicates that contextual background contributed to adolescents' perceptions of parental competence, although the design does not support territorial comparison or population inference. This pattern supports a relational interpretation in which perceived parental competence is linked to both positive resources and distress indicators, rather than to a single continuum of symptom severity.

The results allow us to interpret perceived parental competence as a relational indicator closely linked to how adolescents evaluate their well-being and cope with emotional demands. The strongest positive association was observed with life satisfaction (r = 0.362, *p* < 0.001), followed by resilience (r = 0.283, *p* < 0.001), while the strongest negative association was observed with risk-oriented suicidal ideation (r = −0.354, *p* < 0.001), followed by depressive symptoms (r = −0.278, *p* < 0.001). This pattern is consistent with a two-factor understanding of mental health, in which perceived parenting is linked not only to lower symptom burden but also to the presence of positive psychological resources.

The PANSI operationalization is central to the interpretation of the study. Using an untransformed total score would aggregate negative suicidal ideation and protective ideation in the same direction, producing an ambiguous indicator. After protective items were reverse-coded and combined with negative ideation items, higher PANSI values consistently represented greater suicide-related risk and lower protective ideation. Under this specification, higher perceived parental competence was associated with lower risk-oriented suicidal ideation, which is theoretically coherent and avoids the misleading implication that perceived parental competence is positively related to suicide risk. Even with this correction, the cross-sectional design prevents determining whether perceived parental competence reduces risk, whether lower distress improves perceptions of parenting, or whether both are shaped by unmeasured family and school conditions.

The CRAFFT-related finding requires a different interpretation. The variable was analyzed as a continuous derived screening score, not as the standard categorical CRAFFT cutoff. It showed a small positive bivariate association with perceived parental competence but was not retained in the final regression model, suggesting that this association may overlap with other variables or reflect contextual exposure, disclosure, or monitoring processes rather than an independent effect. Accordingly, this finding should not be interpreted as evidence of association with validated CRAFFT risk classification or substance-related disorders.

For suicide-related ideation, aligning score direction according to the instrument structure strengthened interpretability and produced a clinically coherent association. For substance-use screening, the available coding limits inference because it indexes relative endorsement in this dataset rather than a standard risk category. Together, these results indicate that perceived parental competence is most consistently related to life satisfaction, resilience, depressive symptoms, and risk-oriented suicidal ideation, whereas evidence involving substance-use screening remains exploratory.

These findings are consistent with regional and global studies that highlight the relevance of perceived parenting to adolescent well-being. Globally, evidence has shown that parental emotional support is associated with higher levels of adolescent well-being, and that perceived parental support, supervision, and availability may be related to less loneliness, greater self-regulation, better psychological adjustment, and greater life satisfaction (5–7, 26). In Colombia, parenting styles characterized by greater affection and balanced control have been associated with lower levels of school stress, while control without affection and neglect have been linked to greater academic distress (3). Therefore, the interpretation remains restricted to adolescents' perceptions of parental support, responsiveness, structure, and monitoring, rather than broader claims about observed parenting practices or parent-reported parental competence.

In Latin America, early bonding, parental sensitivity, and attachment are relevant dimensions for understanding child and adolescent development, although regional evidence remains concentrated in urban and middle-class samples (9). Cross-cultural literature on parenting also indicates that parenting practices take on different meanings depending on the context; however, the combination of warmth, communication, structure, and sensitivity tends to be associated with better trajectories of psychological adjustment (10, 21). The results are also consistent with evidence from low- and middle-income countries, where parental and family interventions have shown promising effects on child and adolescent mental health, along with persistent gaps regarding cultural adaptation, mechanisms of change, and community implementation (15, 25).

This study provides empirical evidence on perceived parental competence as a construct located at the intersection of dual-factor models of mental health, ecological approaches to development, and relational theories of parenting. The findings support the relevance of a multidimensional understanding of adolescent mental health, as perceived parental competence was associated with both positive indicators, such as life satisfaction and resilience, and symptomatic indicators, particularly depressive symptoms. This pattern reinforces the need to examine adolescent mental health beyond the mere absence of psychopathology.

The results also support an ecological interpretation of the phenomenon. The inclusion of origin in the model suggests that perceptions of parenting are not shaped independently of the social and territorial context, although the study did not aim to compare territorial groups. The findings also align with approaches to parenting styles and perceived parenting by showing that adolescents' perceptions of caregiver support, availability, and competence may be linked to coping resources, agency, and a positive evaluation of life. Perceived parental competence may therefore be understood as a relevant relational indicator for examining why some adolescents report greater well-being resources in the presence of risk conditions, whereas others report greater emotional symptomatology. This contribution is particularly relevant for Colombian and Latin American contexts, where evidence on parenting and adolescent mental health remains limited and often comes from less heterogeneous samples.

The study has clinical, school-based, and public health implications. The results suggest that perceived parental competence may be incorporated into adolescent mental health assessments as a relational indicator for identifying protective resources and potential sources of vulnerability. In school settings, interventions should not be limited to screening for depressive symptoms, substance use, or suicide risk; they may also include components aimed at strengthening family communication, perceived support, normative consistency, emotional availability, and caregivers' capacity to respond to adolescents' developmental needs. The association with life satisfaction and resilience further suggests that preventive strategies may benefit from a well-being promotion approach, rather than focusing solely on risk reduction. Policy and practice implications are therefore framed cautiously. The findings support including adolescent perceptions of family support and responsiveness in school and community assessments, but they do not demonstrate that modifying parental competence would directly reduce depression, suicide risk, or substance use.

The results also suggest that school-based assessment should distinguish between relational perceptions, emotional symptoms, suicide-related ideation, and screening scores for substance use rather than treating them as interchangeable indicators of distress. The negative association between perceived parental competence and risk-oriented suicidal ideation supports including adolescents' appraisal of parental support and monitoring in school and community assessments. However, the continuous CRAFFT-related result should be used only to generate hypotheses about disclosure, monitoring, and contextual exposure, not to identify categorical substance-use risk or infer intervention effects.

This interpretation is especially relevant in Colombia and other Global South contexts, where the family may serve as a source of protection in the face of inequality, barriers to service access, exposure to violence, academic pressure, and territorial transformations. At the same time, family dynamics may amplify distress when neglect, invalidation, conflict, or insensitive control predominate. These findings support the need for school- and community-based models that work in coordination with adolescents, families, and institutions, while avoiding placing responsibility for mental health solely on the individual.

This study has several limitations. The cross-sectional design precludes establishing causal relationships or temporal directionality between perceived parental competence, well-being, and mental health symptoms. Lower perceived parental competence may contribute to adolescent distress; however, depressive symptoms, suicide risk, or school-related stress may also shape how adolescents interpret parenting practices. In addition, all variables were self-reported, which may introduce recall bias, social desirability bias, effects of current emotional state, or differential interpretation of items.

Although the sample included adolescents from urban, semi-urban, and rural contexts to increase heterogeneity, it was not designed to establish representative territorial comparisons or to explain deep contextual differences. The risk-oriented PANSI operationalization improved interpretability by aligning opposite-valence items into a clinically coherent score, but the cross-sectional and self-reported nature of the data still prevents causal or temporal inference. In contrast, the CRAFFT-related score remains more limited because it was analyzed as a continuous derived screening measure rather than as a standard categorical risk indicator; therefore, future studies should apply validated CRAFFT cutoff scoring, examine CPPA-1 subdimensions, and use longitudinal, multilevel, and mixed-methods designs that integrate adolescent, caregiver, and teacher perspectives. Moreover, although the model explained 26.4% of the variance, a substantial proportion of perceived parental competence remained unexplained, suggesting the need to incorporate family communication, intrafamilial conflict, household structure, caregiving practices, parenting styles, social support, exposure to violence, caregivers' mental health, and socioeconomic conditions.

## Conclusions

This study provides evidence on the correlates of youth-perceived parental competence in Colombian adolescents. Higher perceived parental competence was mainly associated with greater life satisfaction, higher resilience, higher frustration tolerance in the adjusted model, fewer depressive symptoms, and lower risk-oriented suicidal ideation. Because perceived parental competence was modeled as the dependent variable in a cross-sectional design, these findings should be interpreted as associations rather than causal effects of parenting on mental health.

The multivariable model explained 26.4% of the variance in perceived parental competence. More specifically, the final model retained life satisfaction, origin, resilience, risk-oriented suicidal ideation, depressive symptoms, and frustration tolerance. The continuous CRAFFT-related score was not retained in the final model and should be interpreted only as an exploratory screening indicator, not as a validated categorical risk classification. Family-related implications should therefore remain framed as recommendations for assessment and hypothesis generation, not as evidence of causal intervention targets.

## Data Availability

The raw data supporting the conclusions of this article will be made available by the authors, without undue reservation.
